# βTrCP is Required for HIV-1 Vpu Modulation of CD4, GaLV Env, and BST-2/Tetherin

**DOI:** 10.3390/v10100573

**Published:** 2018-10-19

**Authors:** Yul Eum Song, Daniel Cyburt, Tiffany M. Lucas, Devon A. Gregory, Terri D. Lyddon, Marc C. Johnson

**Affiliations:** 1 Department of Molecular Microbiology and Immunology, University of Missouri, School of Medicine and the Christopher S. Bond Life Sciences Center, Columbia, MO 65211, USA; ysqb6@mail.missouri.edu (Y.E.S.); dwcxc5@mail.missouri.edu (D.C.); virologistD@gmail.com (D.A.G.); lyddontd@missouri.edu (T.D.L.); 2 Gene Therapies Branch, Center for Biologics Evaluation and Research, FDA, 10903 New Hampshire Ave, Silver Spring, MD 20903, USA; lucastiffanym@gmail.com

**Keywords:** HIV-1, Vpu, CD4, GaLV Env, BST-2/tetherin, Cullin1, βTrCP

## Abstract

The Human immunodeficiency virus-1 (HIV-1) accessory protein Vpu modulates numerous proteins, including the host proteins CD4 and BST-2/tetherin. Vpu interacts with the Skp, Cullin, F-Box (SCF) ubiquitin ligase through interactions with the F-Box protein βTrCP (1 and/or 2). This interaction is dependent on phosphorylation of S^52,56^ in Vpu. Mutation of S^52,56^, or inhibition of the SCF, abolishes most Vpu activity against CD4 and partly reduces activity against BST-2/tetherin. Recently, Vpu has also been reported to interact with the clathrin adapter proteins AP-1 and AP-2, and these interactions were also found to be required for BST-2/tetherin antagonism in an S^52,56^ -dependent manner. In assays where HIV-1 is pseudotyped with gibbon ape leukemia virus (GaLV Env), Vpu has also been found to prevent GaLV Env from being incorporated into viral particles, but the mechanism for this antagonism is not fully understood. To clarify the role of the βTrCPs in Vpu function we used CRISPR/Cas9 to generate a clonal cell line lacking both βTrCP-1 and -2. Vpu activity against CD4 and GaLV Env was abolished in this cell line, and activity against BST-2/tetherin reduced significantly. Mutation of the S^52,56^ residues no longer affected Vpu activity against BST-2/tetherin in this cell line. These data suggest that the primary role of the S^52,56^ residues in antagonism of CD4, GaLV Env, and BST-2/tetherin is to recruit the SCF/βTrCP ubiquitin ligase.

## 1. Introduction

Human immunodeficiency virus-1 (HIV-1) Vpu is a small 81–86 amino acid accessory protein that enhances viral replication through the antagonism of a number of host cell proteins during infection [1,2]. Vpu has two key features that dictate functionality: an N-terminal transmembrane anchor that is capable of interacting with other transmembrane proteins, and a C-terminal cytoplasmic tail which contains two critical serine residues (S^52,56^) that are phosphorylated and can dictate interaction with various cellular proteins. The best studied targets of Vpu are CD4, which is the receptor for HIV-1, and BST-2/tetherin, an interferon-induced restriction factor which can prevent viral egress [3,4,5]. Antagonism of CD4 by Vpu occurs at the endoplasmic reticulum (ER) through interactions in the cytoplasmic tails of the two proteins [4]. Early studies demonstrated that the membrane anchor sequence of Vpu could be scrambled without compromising the ability of Vpu to antagonize CD4, but changes to the cytoplasmic tail, particularly S^52,56^, abolished activity [4,6,7,8,9]. A cellular Skp, Cullin, F-Box (SCF) E3 ubiquitin ligase complex is recruited by Vpu to target CD4 for ubiquitination and proteasomal degradation [10]. The specific SCF complex utilized by Vpu contains one of two closely related F-box proteins, βTrCP-1 (BTRC) or βTrCP-2 (FBXW11) [11,12]. Vpu antagonism of BST-2/tetherin, on the other hand, is more nuanced as antagonism occurs through multiple steps [3,5,13,14,15]. First, Vpu directly interacts with BST-2/tetherin through its transmembrane domain. This interaction is sufficient to partially displace BST-2/tetherin from the virus assembly site without downmodulating it from the cell surface, but additional domains in Vpu are required to fully counteract BST-2/tetherin [13,15]. Second, Vpu downmodulates BST-2/tetherin from the cell surface by preventing the trafficking of BST-2/tetherin to the plasma membrane from the trans Golgi network and/or the recycling endosome. BST-2/tetherin is constitutively endocytosed from the plasma membrane in a clathrin and adapter protein-2 (AP-2) dependent manner, but the rate of this endocytosis is not altered by Vpu [14,16,17,18]. The surface downmodulation activity requires both the transmembrane anchor and the S^52,56^ residues of Vpu. While it is generally agreed that Vpu retains some ability to counteract tetherin in the absence of S^52,56^ [5,19,20], it is disputed what cellular co-factors Vpu recruits through S^52,56^ to facilitate surface downmodulation. While some reports suggest that βTrCP-1 and -2 are partially or entirely required for this activity, others suggest that the adapter proteins AP-1 and/or AP-2 are the primary co-factors [14,18,19,20,21,22,23,24,25]. In addition, treatment of Vpu expressing cells with MLN4924, a compound that blocks SCF activity, does not restore surface BST-2/tetherin expression unless cells are first treated with interferon, suggesting that the relevant cellular cofactors vary depending on expression level [26,27]. Finally, Vpu targets BST-2/tetherin for degredation. Though early accounts suggested that BST-2/tetherin is proteasomally degraded [23,28] most reports find that the primary mechanism for degradation is through the lysosome [14,16,19,24,29,30,31,32]. It is generally agreed that BST-2/tetherin degradation requires both the transmembrane anchor and S^52,56^ and is SCF/βTrCP mediated [14,19,20,21,23], but it is not clear how important degradation is to overall activity. Indeed, in COS cells Vpu enhances the steady state levels of BST-2/tetherin while simultaneously counteracting BST-2/tetherin antiviral activity [33].

We and others identified a novel target for Vpu which is the surface viral glycoprotein from gibbon ape leukemia virus (GaLV Env) [34,35]. GaLV Env was found to be capable of pseudotyping HIV-1 particles when minimal packaging vectors were used (lacking accessory genes), but could not generate infectious pseudotyped particles with vectors that included Vpu. In the presence of Vpu, GaLV Env is prevented from being incorporated into particles, resulting in a 50–100 fold reduction in infectious particle production [35]. In many ways Vpu appears to modulate GaLV Env using a mechanism that is similar to the modulation of CD4. Like the modulation of CD4, Vpu variants with the membrane anchor sequence scrambled are still able to modulate GaLV Env, but mutation of the S^52,56^ residues abolishes activity [35,36]. Also like with CD4, the Vpu recognition sequence in GaLV Env resides in the cytoplasmic tail, and the amino acid motif required for Vpu modulation closely resembles the motif found in the CD4 cytoplasmic tail [37]. Interestingly, GaLV Env is a trimer and it was shown using functional complementation that GaLV Env can only be targeted by Vpu if all three subunits contain their cytoplasmic tail, even though the three tails do not all have to contain the Vpu recognition motif [38]. Despite the similarities to CD4 modulation, in other ways the modulation of GaLV Env seems to use a mechanism similar to the modulation of BST-2/tetherin. While CD4 is believed to be modulated in the endoplasmic reticulum (ER), BST-2/tetherin is modulated from post-ER compartments. GaLV Env is cleaved by a cellular protease in the Golgi apparatus to reach its mature form, and it is only the mature form that appears to be modulated by Vpu, suggesting that the modulation, like with BST-2/tetherin, occurs in a post-ER compartment [34,37]. In addition, like with BST-2/tetherin, Vpu directs degradation of GaLV Env in the lysosome rather than the proteasome [34]. Also like with BST-2/tetherin, Vpu is capable of preventing the incorporation of GaLV Env into viral particles without reducing the GaLV Env surface expression [35]. Finally, unlike Vpu modulation of CD4 or BST-2/tetherin, GaLV Env modulation by Vpu was reported to require additional HIV-1 structural components [34].

Because Vpu has a strongly negative, rather than positive, impact on HIV-1 infectivity with GaLV Env, this combination could be adapted for use as a gain-of-function screen for inhibitors of Vpu. However, if such a screen is to be utilized, it is important to understand the mechanism of Vpu activity against GaLV Env compared to the other known Vpu targets. In this study, we sought to further characterize the mechanism of GaLV Env modulation by Vpu in comparison to CD4 and BST-2/tetherin. In particular, to examine the importance of βTrCP-1 and -2 in the modulation of these proteins by generating a novel cell line using CRISPR/Cas9 technology where both proteins were eliminated. This unique tool allowed us to definitively show that βTrCP is required for Vpu modulation of CD4, GaLV Env, and BST-2/tetherin.

## 2. Materials and Methods

### 2.1. Plasmids

NL4-3-derived HIV-CMV-GFP was provided by Vineet KewalRamani (National Cancer Institute (NCI)—Frederick) and has been described previously [35]. This proviral vector lacks the accessory genes Vif, Vpr, Nef, and Env and contains a CMV promoter-driven GFP in place of Nef. We used versions of this vector both with and without the Vpu gene (Vpu^+/−^). For imaging studies, a nuclear localization sequence (KKKRK) was fused to the N-terminus of the GFP sequence to allow easier enumeration of infected cells (HIV-CMV-NLS-GFP).

The ecotropic Friend MLV Env (isolate 57) expression construct and a subclone of this construct containing YFP in the protein’s ectodomain were provided by Walther Mothes (Yale University) [39]. The MLV/GaLV Env ∆8 and ∆12 constructs were described previously [35,37]. Derivatives of these constructs were generated with a hemagglutinin (HA) tag or mCherry tag in place of the YFP sequence. The cherry tagged GaLV Env ∆8 was subcloned into retroviral transfer vector PQCXIH (Clontech, Mountain View, CA, USA) for generation of a stable cell line. The pSpCas9(BB)-2A-Puro (PX459) V2.0 was a gift from Feng Zhang (Addgene plasmid # 62988) [40]. The βTrCP-1 and -2 guide RNA (gRNA) sequences (GTAATAATGGCGAACCCCCT, and GACACGGCGTAAAGTGATGT, respectively) were synthesized, and inserted into the *BsmB*I restriction site in PX459. The Ub-K0 construct was provided by Mark Hannink (University of Missouri). The dominant negative (DN) βTrCP and the Vpu-GFP constructs were provided by Ed Stephens (University of Kansas Medical Center) [14,41]. The pHAGE DN Cullin1 was a gift from Stephen Elledge (Addgene plasmid # 41911) [42]. Vpu-IRES-GFP and pcDNA3.1 human CD4 plasmids were kindly provided by Frank Kirchoff (University of Ulm, Germany) [43]. For western blotting, hemagglutinin (HA) tag (YPYDVPDYA) was inserted in the N-terminal of CD4 immediately after the signal peptide. Human Tetherin-HA plasmid was kindly provided by Paul Bieniasz [44]. The retroviral transfer vector for stably expressing human βTrCP-2 was obtained by amplifying βTrCP-2 from human cDNA collected from 293FT cells cloning it into vector pQCXIH (Clontech).

### 2.2. Cell Lines

The 293FT cell line was obtained from Invitrogen (Carlsbad, CA, USA). This cell line is a HEK293 derivative similar to 293T, which has been adapted for high transfectability. The βTrCP double knockout cell lines were obtained by transfecting the 293FT cells with PX459 plasmids encoding βTrCP1 and βTrCP2 gRNA described in plasmids section. Single cell isolates from the transfected population were screened by transfecting clonal isolates with HIV-CMV-GFP (Vpu^+^) and GaLV Env and testing if infectious particles are produced. Isolates that were able to produce infectious particles under these conditions were further screened by amplifying and sequencing the sequence flanking the Cas9/gRNA target site. βTrCP1/2 knockout cells with βTrCP2 add back were obtained by transducing the knockout cell line with an MLV based vector coding for both a hygromycin resistance gene and βTrCP2 (amplified from 293FT cell cDNA); the transduced population was subsequently selected with hygromycin. The mCherry tagged-GaLV Env ∆8 expression cell line was produced the same way except 293 TVA cells were used [45]. These cells were chosen because they are a cell line our lab routinely uses which are morphologically more suitable for imaging studies than 293FT cells. While both cell lines are HEK293 derivatives, 293FT cells tend to grow in clumps and adhere poorly to coverslips, whereas 293 TVA cells (like 293T cells) appear much flatter and adhere to coverslips much more firmly. The difference is not believed to be a result of the TVA transgene as most HEK293 derivatives, with the exception of 293FT, have a morphology similar to 293 TVA cells. Following selection, cells were sorted using a Beckman Coulter MoFlo XDP (Cell and Immunobiology Core, University of Missouri) for cells that express high levels of mCherry. This cell population was transduced with HIV-CMV-NLS-GFP (Vpu^+/−^) at a relatively low (<2) multiplicity of infection (MOI). The cell line expressing the ecotropic F-MLV Env receptor, 293T mCAT-1, was provided by Walther Mothes. All cell lines were maintained in Dulbecco’s modified Eagle media (DMEM), containing 10% fetal bovine serum, 2 mM l-glutamine, 1 mM sodium pyruvate, 10 mM nonessential amino acids, and 10 mM MEM vitamins.

### 2.3. Western Blotting

For inhibitor studies on protein expression, 293FT cells were transfected with 500 ng HA-tagged GaLV Env ∆8, 25 ng Vpu-GFP and 200 ng empty plasmid. At 16 h post-transfection, cells were treated for 7 h with either a DMSO control, proteasomal inhibitor cocktail (10 µM MG132 and 20 µM epoximicin) or a lysosomal inhibitor (100 nM Bafilomycin A1). Cellular 293FT lysate was collected and treated with PNGase as previously described [34]. Lysate protein concentrations were determined using a Bradford reagent against a BSA standard and 10 µg of protein was resolved by SDS-PAGE and immunoblotted with anti-HA (Sigma H3663, 1:1000, St. Louis, MO, USA) followed by anti-mouse HRP (Sigma A5278, 1:10,000). Following transfer, blots were stained with Ponceau S to confirm protein transfer and equivalent loading. For CD4 expression, 293FT cells and its derivatives were transfected with 100 ng of HA-tagged CD4 and 500 ng of either Vpu-IRES-GFP or 500 ng IRES-GFP. Cellular lysate was collected after 2 days and 15% of whole cell lysate was resolved by SDS-PAGE and immunoblotted. CD4 expression was detected by anti-HA, and GAPDH was detected for loading control (Santa Cruz Biotechnology SC-47724, 1:500, Dallas, TX, USA).

### 2.4. Infectivity Assays

Except where noted, infectivity assays were performed by transfecting 293FT cells or its derivatives with the Env defective HIV-CMV-GFP proviruses. All transfections were performed in 35 mm dishes using polyethylenimine (PEI) [46]. Virus containing media was frozen at −80 °C prior to infections in 293T mCAT-1 cells. Transduced cells were fixed with paraformaldehyde two days post-transduction, and analyzed on an Accuri flow cytometer. For proteasomal and lysosomal inhibitor studies, cells were transfected with 600 ng of HIV-CMV-GFP (Vpu^+/−^) and 400 ng GaLV Env ∆8 or GaLV Env ∆12. Cells were treated 40 h post-transfection with either proteasomal inhibitors (20 µM ALLN or 10 µM MG132), lysosomal inhibitors (100 nM Bafilomycin A1 (BafA) or 100 nM Concanamycin A (ConA)), a DMSO control, or no treatment (NT). Virus-containing culture media was collected 12 h later and spinoculated onto 293T mCAT-1 cells at 30 °C for 30 min. For infectivity with the mCherry-GaLV Env ∆8 transduced cell line, both the virus producing cells and the virus containing media were collected. The cells were fixed and analyzed using an Accuri flow cytometer to determine the number of transduced (GFP-expressing) cells and the viral media was used to transduce 293T mCAT-1 cells. The output was expressed as the number of infectious units per 10^6^ virus producing cells. For polyubiquitin experiments, cells were transfected with 250 ng of HIV-CMV-GFP (Vpu^+/−^), 250 ng GaLV Env ∆8 or GaLV Env ∆12, and 500 ng dominant-negative ubiquitin (Ub-K0). For Neddylation inhibitor studies, cells were transfected with 900 ng of provirus and 100 ng of MLV Env or GaLV Env ∆8. The next day, cells were treated with 1 μM MLN4924 and viral media was collected 24 h later. For DN βTrCP and DN Cullin1 studies, cells were transfected with 500 ng of provirus, 100 ng of MLV Env or GaLV Env ∆8 and 500 ng or 100 ng of DN βTrCP and DN Cullin1, respectively. For experiments in the knockout cell lines, cells were transfected with 900 ng of HIV-CMV-GFP (Vpu^+/−^), 100 ng of MLV Env or GaLV Env ∆8. For BST/tetherin activity assays, cells were transfected with 900 ng of HIV-CMV-GFP (Vpu^+/−^), 100 ng of VSV-G and 0–80 ng of BST-2/tetherin.

### 2.5. Surface Labeling

For CD4 surface labeling, 293FT cells and derivatives were plated in 35-mm dishes and transfected the following day with 100 ng human CD4 and either 500 ng Vpu-IRES-GFP or 500 ng IRES-GFP. Where appropriate, wells were transfected with varying amounts of DN βTrCP (500 ng) or DN Cullin1 (100 ng). For experiments employing MLN4924, media was changed 24 h post-transfection and replaced with media containing 1 µM MLN4924 in DMSO; surface staining was performed 24 h post-media change. For surface staining, cells were lifted from the plates using PBS with 10 mM EDTA, blocked with 5% goat serum in PBS for 30 min, and incubated in 96 well U-bottom plates for 1 h with 1% anti-CD4 APC conjugate primary antibody in PBS/EDTA with 1% goat serum. After staining, cells were washed with PBS and fixed with 1% paraformaldehyde. Surface staining of transfected cells was analyzed the same day on an Accuri C6 flow cytometer. CD4 surface staining was read as the mean fluorescence intensity of the far-red APC signal among the GFP-transfected cell population. For mCherry-GaLV Env ∆8 surface labeling, cells were first blocked with 5% goat serum in PBS for 30 min, incubated with primary anti-mCherry rabbit antibody (abcam ab167453), and diluted 1:500 in PBS for 1 h at 4 °C. After incubation, cells were washed 2–3 times with PBS and fixed in 4% paraformaldehyde for 20 min, and washed again 2–3 times with PBS. Cells were then blocked again with 5% goat serum in PBS for 30 min and incubated in secondary anti rabbit Alexa 405 antibody (Life Technologies, Carlsbad, CA, USA) in 10 µg/mL concentration for 1 h at 4 °C. Cells were finally washed with PBS 2–3 times and analyzed on flow cytometer (BD LSRFortessa X-20, (San Jose, CA, USA) at the Cell and Immunobiology Core at the University of Missouri, and analyzed with flowJo software (version 10.3, FlowJo, LLC, Ashland, OR, USA).

### 2.6. Imaging

For mCherry-GaLV Env ∆8 imaging, cells were plated on a glass bottom dish (MatTek, Ashland, MA, USA). Cells were first blocked with 5% goat serum in PBS for 30 min, incubated with primary anti-mCherry rabbit antibody (abcam ab167453), and diluted 1:500 in PBS for 1 h at 4 °C. After incubation, cells were washed 2–3 times with PBS and fixed in 4% paraformaldehyde for 20 min, and washed again 2–3 times with PBS. Cells were then blocked again with 5% goat serum in PBS for 30 min and incubated in secondary anti rabbit Alexa 405 antibody (Life Technologies) in 10 µg/mL concentration for 1 h at 4 °C. Cells were finally washed with PBS 2–3 times. Cells were imaged on an Olympus IX81 with 100 × 1.4 oil objective with deconvolution processing of Olympus CellSens Dimension software (version 1.3, Olympus, Shinjuku, Tokyo, Japan). Images were adjusted using ImageJ software to highlight the different intensity of signal.

## 3. Results

The glycoprotein used for analyzing the mechanism of Vpu action in this study is a chimeric ecotropic MLV Env glycoprotein that contained the C-terminal cytoplasmic tail from GaLV Env with the last 8 amino acids deleted (henceforth referred to as GaLV Env ∆8). This glycoprotein was chosen because we showed previously that it forms infectious particles with HIV-1 much more efficiently than wildtype GaLV Env, but is still potently prevented from being incorporated into HIV-1 particles by Vpu [37]. Infectious HIV-1 particle production with GaLV Env ∆8 correlates with incorporation into viral particles, making loss of infectious particle production a quantitative measure of Vpu activity.

### 3.1. GaLV Env ∆8 Is Targeted for Lysosomal Degradation

**Figure 1:**
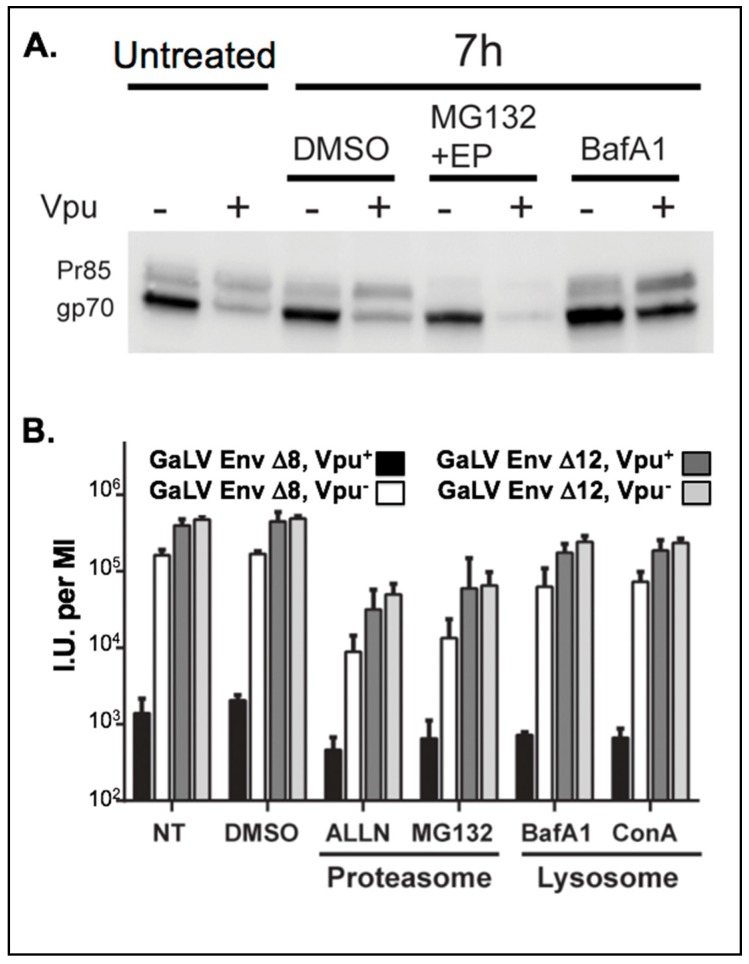
Vpu depletes mature GaLV Env ∆8 in cells. (**A**) 293FT cells were transfected with hemagglutinin (HA)-tagged GaLV Env ∆8 and GFP-tagged Vpu. 16 h post-transfection, cells were treated for 7 h with either a DMSO control, proteasomal inhibitor cocktail containing 10 µM MG132 and 20 µM epoximicin (EP), or the lysosomal inhibitor Bafilomycin A1 (BafA1, 100 nM). Then, 10 µg of cell lysate was immunoblotted with anti-HA antibody. (**B**) 293FT cells were transfected with HIV-CMV-GFP (Vpu^+/−^) and GaLV Env ∆8 or GaLV Env ∆12. At 40 h post-transfection, cells were treated with either proteasomal inhibitors MG132 (10 µM) or ALLN (20 µM), or lysosomal inhibitors BafA (100 nM) or Concanamycin A (ConA, 100 nM), a DMSO control, or no treatment (NT). After 12 h, virus-containing culture media was collected and spinoculated onto 293T mCAT-1. Transduced cells were measured by flow cytometry 48 h later. I.U. = infectious units.

We first sought to evaluate the effect of Vpu on the steady-state levels of GaLV Env ∆8. Cells were transfected with GaLV Env ∆8 along with an independent Vpu expression plasmid and GaLV Env expression in cell lysates was measured by western blot. As reported previously, expression of Vpu did not alter the level of the uncleaved Env precursor protein (Pr85), but greatly reduced the amount of mature cleaved (gp70) Env protein [34,37] (Figure 1A). Because retroviral Env proteins are not cleaved until they pass through the Golgi apparatus [47], this is consistent with GaLV Env ∆8 being targeted by Vpu in a post-ER compartment. HIV-1 Vpu targets CD4 for degradation through proteasomal degradation [10], but targets BST-2/tetherin through lysosomal degradation [14,24]. HIV-1 Vpu was previously reported to mediate lysosomal degradation of GaLV Env [34]. To verify that the GaLV Env construct used in this study behaves the same as the full-length protein used previously, transfected cells were treated with lysosomal or proteasomal inhibitors. Treatment of cells with lysosomal inhibitor Bafilomycin A restored mature Env in cells, but treatment with proteasome inhibitors did not (Figure 1A). These results are consistent with the previous report; however, unlike the previous report [34], we found that Vpu was sufficient to modulate the mature GaLV Env expression level and did not require the presence of additional HIV-1 structural proteins.

### 3.2. Inhibition of Lysosomal Degradation Does Not Restore Infectious Particle Production with GaLV Env ∆8

We next asked whether lysosomal inhibition was sufficient to restore infectious particle production with GaLV Env ∆8. For this experiment, an NL4-3 derived HIV-1 provirus (Vpu^+/−^) lacking Env, Vif, and Vpr and containing CMV-GFP in place of Nef (henceforth HIV-CMV-GFP) was pseudotyped with GaLV Env ∆8, and infectious particle output was measured following treatment with proteasomal or lysosomal inhibitors. As a control, the same proviruses were pseudotyped with GaLV Env ∆12, which has four additional amino acids deleted rendering the protein insensitive to Vpu [37]. As expected, Vpu reduced the infectious particle production with GaLV Env ∆8 by approximately 100-fold, but had no effect on the infectious unit production with Vpu-insensitive GaLV Env ∆12 (Figure 1B). Neither proteasomal nor lysosomal inhibitors restored infectious particle production to HIV-1 particles pseudotyped with GaLV Env ∆8. Proteasomal inhibitors noticeably reduced the production of infectious viral particles, but it did so with both glycoproteins and was not affected by Vpu. Thus, preventing GaLV Env ∆8 degradation was not sufficient to restore infectious particle production. A possible explanation for this is that the GaLV Env protein is sequestered in the lysosome and away from viral particles, even if it is not degraded at the lysosome. A similar phenomenon has been observed with Vpu modulation of BST-2/tetherin in COS cells [33].

### 3.3. Loss of Surface Expression Weakly Correlates with Loss of Infectious Particle Production

**Figure 2:**
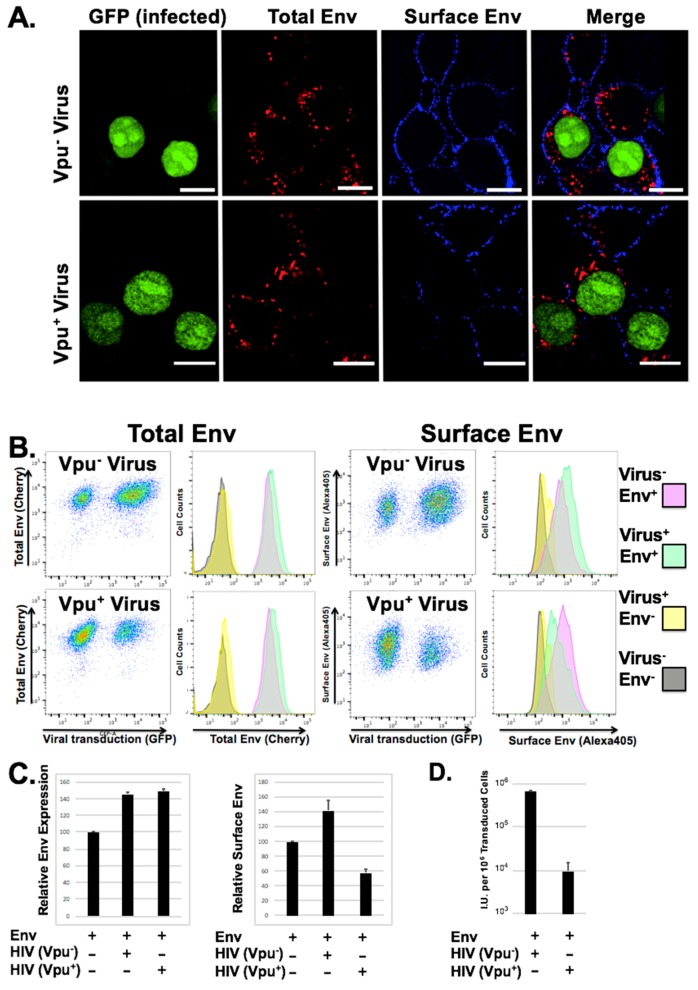
Vpu decreases surface expression of GaLV Env ∆8 and infectious particle production. (**A**) Sorted 293 TVA cells stably expressing mCherry tagged MLV GaLV Env ∆8 were transduced with HIV-CMV-NLS-GFP (Vpu^+/−^) and plated on glass bottom dishes. The presence of GFP (green) indicates that the cell has been transduced. Total Env (cherry) expression is shown in red. Surface Env was labeled with Alexa405 (blue). Scale bars = 10 µM. (**B**) Transduced cells from (**A**) were collected, surface labeled, and analyzed on a flow cytometer for both total and surface MLV GaLV Env ∆8 expression. Transduced cells without Env stably expressed were used as controls (grey/yellow populations) (**C**) Average relative mean fluorescence intensity (MFI) of Env expression and surface Env expression are shown from 3 individual experiments. (**D**) Cells were transduced as explained in (**A**). Supernatants containing VLPs were collected from transduced Env-expressing producer cells (Vpu^+/−^) for infection on 293T mCAT-1. Producer cells were collected and analyzed by flow cytometry to determine the number of virus producing cells. Transduced mCAT-1 cells were measured by flow cytometry 48 h later and infectious unit production (I.U.) per million transduced producer cells was calculated.

We demonstrated previously that loss of infectious particle production with GaLV Env in the presence of Vpu did not require noticeable surface downmodulation [35]. However, these previous studies were performed in transfected cells in which both Vpu and GaLV Env were presumably overexpressed, and the study only addressed surface expression. To study the change in GaLV Env distribution using a system where the protein is not as highly expressed, we first generated a novel cell line expressing a fluorescently tagged version of GaLV Env ∆8 that contained mCherry cloned into the proline rich ectodomain of the glycoprotein. This construct was chosen because it produces infectious particles with similar efficiency to untagged protein, and it remains sensitive to Vpu expression. Next, we transduced these cells at a low MOI (<2) with HIV-CMV-GFP particles (Vpu^+/−^) and analyzed total Env expression, Env distribution, surface Env expression, and infectious particle production (Figure 2). Transduced and un-transduced cells displayed discrete Env punctae within the cytoplasm of cells, but only weak signal at the plasma membrane. There was no obvious difference in quantity or distribution in the mCherry signal in cells transduced with Vpu containing provirus. However, when we stained surface Env expression using an antibody against mCherry, there was a noticeable loss in surface Env expression in Vpu expressing cells relative to neighboring untransduced cells (Figure 2). To quantitate this difference, the cells were collected and analyzed by flow cytometry. There was no quantifiable reduction in total mCherry expression in cells expressing Vpu. However, when surface labeling was quantified there was a consistent 2–3 fold reduction in surface expression relative to cells transduced with virus lacking Vpu. It was surprising that Vpu did not cause a quantifiable difference in overall GaLV Env expression levels, which is in contrast to what we and others have observed in experiments where GaLV Env is transfected into cells. We believe that the difference is because in the stable cells Env is expressed at low level, which may make the Vpu modulation less apparent. Alternatively, the difference could simply reflect differences in the two assays. While western blot (Figure 1A) detects absolute protein levels, the flow cytometry readings (Figure 2B) detect the fluorescent signal from the produced protein. To determine the infectious pseudotyped particle production from these cells, the supernatant was collected and used to transduce fresh cells. Because the Env expressing cells do not express the mCAT-1 receptor, infectivity could only be scored on a secondary cell line that expresses the ecotropic MLV Env receptor mCAT-1 (293T mCAT). While both transduced cell lines produced infectious particles, the output from cells transduced with the Vpu-containing provirus was 50–100 times lower (Figure 2D). Thus, while Vpu only partially downmodulates GaLV Env ∆8 from the surface of cells, it almost completely abrogates production of infectious particles.

### 3.4. Vpu Inhibition of GaLV Env ∆8 Infectious Particle Production Is Polyubiquitin Dependent

**Figure 3:**
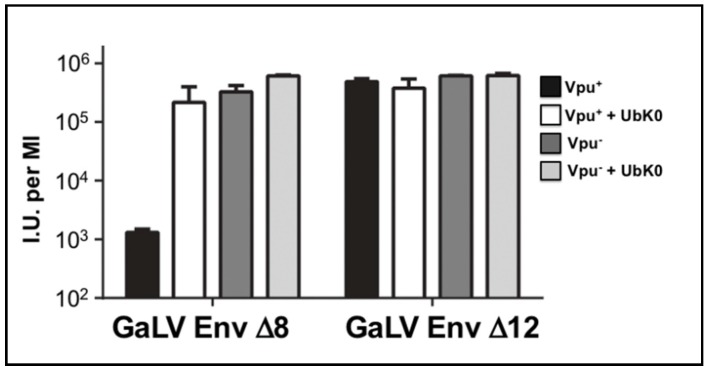
Vpu constrains GaLV Env envelope pseudotyping in a polyubiquitin-dependent manner. 293FT cells were transfected with Vpu sensitive GaLV Env Δ8 or insensitive GaLV Env Δ12, HIV-CMV-GFP and a DN ubiquitin (Ub-K0) expression construct. After 48 h, media was transferred to 293T mCAT-1 cells and infectivity was measured by flow cytometry 48 h later. Bars are mean ± SD for three experimental replicates.

Because some Vpu activities involve polyubiquitination, we employed a dominant negative ubiquitin to address whether polyubiquitination is required to restrict infectious particle production. This dominant negative protein has all seven of its lysine residues mutated to arginine (Ub-K0) such that the protein can be covalently added to substrates, but cannot form ubiquitin chains due to its lack of lysine acceptor residues [48,49,50,51,52]. We found Ub-K0 enhanced infectious particle output of HIV-CMV-GFP (Vpu^+^) approximately 100-fold with GaLV Env ∆8, but it had no effect on HIV-CMV-GFP (Vpu^−^) particles (Figure 3). Ub-K0 had no effect with GaLV Env ∆12 in the presence or absence of Vpu. These findings demonstrate that polyubiquitination is required for the modulation of GaLV Env ∆8 by Vpu.

### 3.5. Vpu Inhibition of GaLV Env ∆8 Infectious Particle Production Is Restored by Neddylation Inhibitor MLN4924

**Figure 4:**
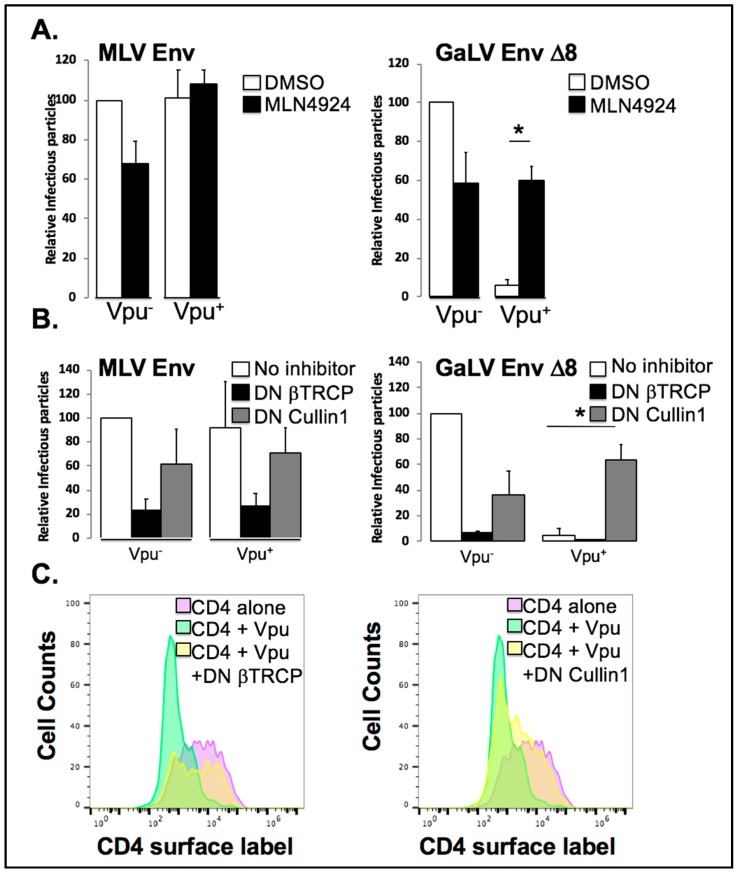
Vpu antagonism of GaLV Env is Cullin1 dependent. (**A**) 293FT cells were transfected HIV-CMV-GFP along with either MLV Env or MLV/GaLV Env Δ8. 24 h post-transfection cells were treated with 1 μM MLN4924, or DMSO. Media was collected after 24 h and was transferred to 293T mCAT-1 cells and infectivity was measured by flow cytometry 48 h later. Infectious particle output was normalized to that of HIV-CMV-GFP (Vpu^−^)/DMSO. (**B**) 293FT cells were transfected with HIV-CMV-GFP along with either MLV Env or MLV/GaLV Env Δ8 and either DN Cullin1, DN βTrCP, or empty vector control. After 48 h, media was transferred to 293T mCAT-1 cells and infectivity was measured by flow cytometry 48 h later. (**C**) 293FT cells were transfected with human CD4 alone, CD4 + Vpu IRES GFP, or CD4 + Vpu IRES GFP + DN Cullin1 or DN βTrCP. CD4 surface protein was labeled and analyzed by flow cytometry 48 h later. Asterisks indicate *p* < 0.01.

The dependence on both polyubiquitin chains (Figure 3) and the Vpu S^52,56^ residues [35] indicated that Vpu may be hijacking and utilizing a ubiquitin ligase to prevent mature GaLV trafficking to viral particles. MLN4924 is an ATP analogue that binds to and irreversibly inhibits the Nedd8 activating enzyme, a member of a pathway responsible for crosslinking a Nedd8 moiety onto Cullin proteins and critically important for their activity [53]. As such, MLN4924 is a strong inhibitor of all Cullin-containing E3 ubiquitin ligases in the cell [54]. To determine if Cullin activity is required for Vpu activity against GaLV Env ∆8, we transfected HIV-CMV-GFP (Vpu^+/−^) along with GaLV Env ∆8 or Vpu-resistant MLV Env, and measured infectious particle output in the presence or absence of MLN4924 (Figure 4A). Because of variation in infectious particle production among glycoproteins, the infectious particle output for each set of experiments is normalized and expressed as a percent compared to the Vpu^−^ provirus with no treatment. Infectious output from particles generated with MLV Env was not altered by Vpu expression and was modestly inhibited by MLN4924. As expected, infectious particle output from cells expressing GaLV Env ∆8 was severely restricted by Vpu. However, this Vpu inhibition was eliminated by MLN4924 treatment. Thus, Vpu antagonism of GaLV Env is dependent on a cellular Cullin-containing ubiquitin ligase enzyme.

### 3.6. Vpu Inhibition of GaLV Env ∆8 Infectious Particle Production Is Restored by Dominant Negative (DN) Cullin 1, But Not a DN βTrCP

Since Vpu is known to hijack SCF-βTrCP, we used two DN proteins to test if Vpu antagonism of GaLV Env ∆8 was dependent on Cullin1 and the F-box proteins βTrCP-1/-2. DN Cullin1 is missing its Rbx domain, through which it binds to and engages the E2 ubiquitination machinery; as such, it binds to the Skp/F-box protein complex but does not support ubiquitin transfer to the target substrate protein. DN βTrCP is missing its F-box domain, through which it contacts the Skp1/Cullin1/E2 ligase machinery complex, so it will bind to substrate proteins and Vpu, but cannot support ubiquitin transfer to the target protein, and therefore blocks Vpu activity with both βTrCP-1 and -2 [11,14]. We co-transfected HIV-CMV-GFP (Vpu^+/−^) with MLV/GaLV ∆8 Env or MLV Env along with DN Cullin1, DN βTrCP or an empty control vector (Figure 4B). Both DN proteins reduced infectious particle production 2–3 fold with MLV Env regardless of Vpu expression, as well as MLV/GaLV ∆8 particles in the absence of Vpu. This is most likely due to off-target effects of the expressed proteins. Once again, infectious particle production with MLV GaLV Env ∆8 was severely restricted (50–100 fold) in the presence of Vpu. Curiously, dominant negative Cullin1 potently rescued MLV/GaLV ∆8 infectious particle production, while DN βTrCP showed no rescue at all (Figure 4B). This experiment was repeated numerous times with both higher and lower DN βTrCP concentrations, but infectious particle rescue was never observed. SCF ligases utilize over 80 possible F-box proteins and these findings raised the possibility that Vpu is capable of hijacking F-box proteins other than βTrCP, expanding the network of potential E3 enzymes it could subvert. To confirm that the DN βTrCP expression construct was functional, we confirmed that it was capable of restoring CD4 surface expression in the presence of Vpu (Figure 4C).

### 3.7. βTrCP Is Required for Vpu Activity

**Figure 5:**
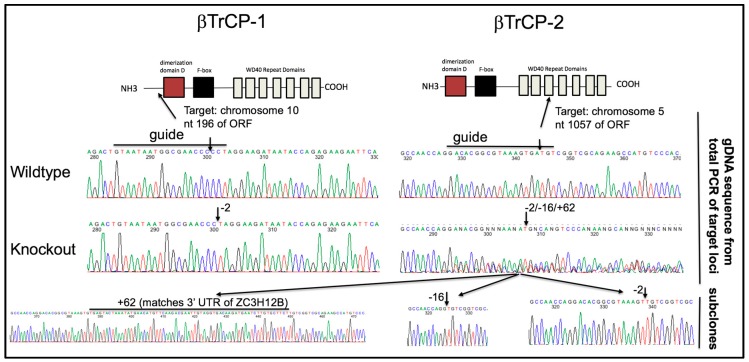
Confirmation of βTrCP-1 and -2 KO. Shown is the schematic of the gRNA target site in the βTrCP-1 and -2 genes. Genomic DNA was extracted from the parent 293FT cell line as well as the βTrCP-1/2 KO cell line. The sequence flanking the CRISPR gRNA target sites was amplified by PCR and sequenced. The sequence in the βTrCP-1 was a homogeneous −2 nt KO. The sequence in the βTrCP-2 locus was appeared to be three distinct variations (−2, −16, +62). This was confirmed by subcloning the PCR and sequencing the three distinct isoforms.

To unambiguously determine the βTrCP-dependency of Vpu activity, we chose to use CRISPR/Cas9 to generate a novel 293FT cell line with both βTrCP-1 and -2 knocked out. CRISPR/Cas9 gRNAs were designed against both genes and introduced into cells. To our surprise, the pool of CRISPR treated cells showed some enhancement in infectious particle production with GaLV Env in the presence of Vpu, suggesting that βTrCP is at least partially required for activity. Clonal isolates were obtained from this population of cells by limiting dilution and cell lines were screened for enhanced infectious particle production with GaLV Env in the presence of Vpu. Because we could not identify any commercial antibodies that reliably detect the two βTrCP proteins, clonal isolates were further screened by recovering the CRISPR gRNA target sites by PCR and sequencing the product to confirm the knockout (KO) at each locus (Figure 5). The clonal isolate selected (βTrCP-1/2 KO) contained a homologous −2 indel in βTrCP-1 locus, and a mixture of −2, −16, +62 indels in the βTrCP-2 locus. These three indels persisted through multiple rounds of clonal isolation, suggesting that the cell line is at least trisomic at this locus, which is on chromosome 5. To rule out any changes in this cell line being a result of off-target CRISPR effects, we stably reintroduced βTrCP-2 to the cell line to examine in parallel (βTrCP-1/2 KO + βTrCP-2, henceforth referred to as “add back”).

**Figure 6:**
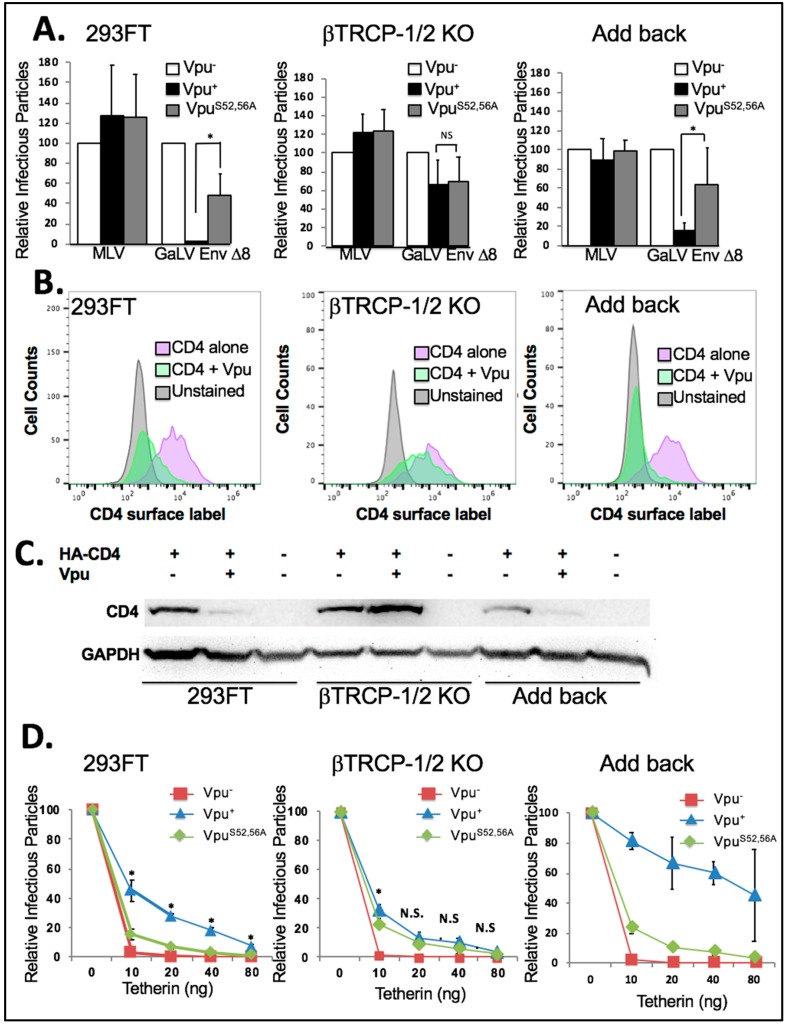
Importance of βTrCP. (**A**) 293FT, βTrCP-1/2 KO, and add back cells were transfected with HIV-CMV-GFP (Vpu^+^, Vpu^−^, and Vpu^S52/56A^) along with MLV Env or GaLV Env Δ8. After 48 h, media was transferred to 293T mCAT-1 cells and infectivity was measured by flow cytometry 48 h later. Infectious particle production from each data set was normalized to HIV-CMV-GFP (Vpu^−^). Shown is the average and standard deviation from six independent experiments. (**B**) The same cell lines were transfected with human CD4 with or without Vpu-IRES-GFP, surface stained for CD4 expression, and analyzed by flow cytometer 48 h later. (**C**) The same cell lines were tranfected with HA-tagged human CD4 with or without Vpu-IRES-GFP, collected after 2 days, and immunoblotted for HA-CD4. GAPDH was blotted as a loading control. (**D**) The same cells lines were transfected with HIV-CMV-GFP (Vpu^+^, Vpu^−^, and Vpu^S52/56A^), along with VSV-G and varying amounts of HA-Tetherin (0–80 ng). After 48 h, media was transferred to 293T mCAT-1 cells and infectivity was measured by flow cytometry 48 h later. Infectious particle production from each data set was normalized to that of the well with 0 ng tetherin. Shown is the average and standard deviation of seven independent experiments. Students *t* test was performed comparing Vpu^+^ to Vpu^S52,56A^. Asterisks indicate *p* < 0.01. NS indicates difference is not significant.

We first tested the βTrCP-1/2 KO and add back cell lines in infectivity assays with HIV-CMV-GFP containing Vpu^−^, Vpu^+^ or Vpu^S52,56A^ along with GaLV Env ∆8 or MLV Env (Figure 6A). Because the three cell lines did not transfect at exactly the same rates, the infectious particle production from each cell line was expressed relative to that from the Vpu^−^ provirus. In both the parental 293FT cell line and the add back cell line the presence of wildtype Vpu greatly reduced infectious particle production (10-fold or more) with GaLV Env ∆8, but Vpu^S52,56A^ had little to no effect (2-fold or less). Surprisingly, we found that in the βTrCP-1/2 KO cell line Vpu lost the ability to restrict GaLV Env ∆8, and the activity of wildtype Vpu was the same as Vpu^S52,56A^. This was in contrast to what was observed with the DN βTrCP (Figure 4B).

Next, we examined the ability of Vpu to downmodulate CD4 in these cell lines. As would be expected, in the parent 293FT cell line and in the add back cell line Vpu greatly reduced surface CD4 expression. In fact, each time the experiment was performed Vpu appeared to downmodulate CD4 more efficiently in the add back cell line than in the parent cell line. This could likely be due to βTrCP-2 expression being higher in the add back cell line than in the parent cell line (Figure 6B). However, in the βTrCP-1/2 KO cell line Vpu was not able to meaningfully downmodulate CD4 expression. When analyzed by western, the steady state levels of CD4 decreased in the presence of Vpu in 293FT and add back cells, but in the βTrCP-1/2 KO cells the CD4 expression level increased in the presence of Vpu (Figure 6C). This confirms that βTrCP is required for CD4 degradation by Vpu.

Finally, we chose to examine the antagonism of BST-2/tetherin by Vpu in these cell lines (Figure 6D). For this experiment each cell line was transfected with HIV-CMV-GFP containing Vpu^−^, Vpu^+^ or Vpu^S52,56A^ and a Vpu-insensitive glycoprotein along with 0, 10, 20, 40, or 80 ng of a tetherin expression plasmid. As expected, in the 293FT parent cell line infectious particle output was essentially abolished with 10 ng of tetherin, but the Vpu^+^ infectious particle output was only partially reduced. The infectious particle output with Vpu^S52,56A^ was higher than with Vpu^−^, but there was a very clear and statistically significant difference between wildtype Vpu and Vpu^S52,56A^. The results were largely the same in the add back cell line except wildtype Vpu appeared to be more effective at countering tetherin than in the parent cell line. This is similar to what was observed in the CD4 modulation experiments and are consistent with βTrCP-2 being able to enhance Vpu activity. The output with the βTrCP-1/2 KO cell line was clearly different. The provirus with Vpu^S52,56A^ was still able to counter tetherin as efficiently as in the parent and add back cell lines, but the provirus with wildtype Vpu had greatly diminished activity. There was a small (0.5-fold) but statistically significant enhancement of wildtype Vpu activity over Vpu^S52,56A^ at 10 ng of tetherin, but at all other concentrations there was no statistically significant difference between wildtype Vpu and Vpu^S52,56A^. We conclude that in this cell line, the predominant function of the S^52,56^ residues in Vpu is to recruit βTrCP.

## 4. Discussion

Here we verified the role of the SCF/βTrCP1/2 E3 ubiquitin ligase as a critical cofactor for Vpu to block incorporation of mature, functional GaLV Env into pseudotyped HIV-1 particles. We further confirmed that this ligase is also critical for Vpu to downmodulate CD4 from the cell surface and to antagonize the host restriction factor BST-2/tetherin. While there has generally been a consensus that βTrCP is the critical host factor required to promote CD4 downmodulation, the role of SCF/βTrCP in BST-2/tetherin antagonism has been more controversial [20,21,26]. Initial studies on the mechanism of how Vpu blocks GaLV Env incorporation into virions suggested that it was at least similar to the CD4 mechanism, since the two proteins share the same Vpu sensitivity motif in the cytoplasmic tail [37]. However, the dominant negative version of βTrCP, which blocked Vpu antagonism of CD4, failed to block its activity against GaLV Env. Therefore the finding that Vpu activity against GaLV Env was lost in βTrCP-1/2 KO cell line was surprising, but clearly showed that βTrCP was required for Vpu to block formation of GaLV Env pseudotypes. This is not the first report of the DN βTrCP yielding a different outcome than knocking down βTrCP-1 and -2. Tervo et al. demonstrated that DN βTrCP could restore CD4 and BST-2/tetherin surface expression in the presence of Vpu, but it could not restore overall BST-2/tetherin expression levels [20]. In contrast, when they knocked down βTrCP-1 and -2 in the presence of Vpu it restored BST-2/tetherin expression levels. The authors speculated that the DN βTrCP may impair the Vpu-dependent proteasomal degradation of Vpu targets, but may not be able to prevent these targets from trafficking to the lysosome. The simplest explanation for the failure of DN βTrCP to restore GaLV Env infectious particle production in the presence of Vpu is that DN βTrCP has off-target effects that distort the assay. In particular, we found that the concentrations of DN βTrCP required to restore CD4 surface expression in the presence of Vpu inhibited infectious particle production even in the absence of Vpu (Figure 4). In the data shown, the same concentration of DN βTrCP was used in both the CD4 and GaLV Env assays. Lower concentrations of DN βTrCP was less detrimental to infectivity, but was also less effective at restoring CD4 expression in the presence of Vpu. DN βTrCP was unable to enhance GaLV Env infectivity in the presence of Vpu at any of the concentrations.

While there has been controversy on the role of βTrCP in antagonism of BST-2/tetherin by Vpu, the studies refuting its importance have usually employed siRNA knockdown of the βTrCPs, while this is to our knowledge the first time these proteins have been permanently knocked out in virus producing cells [20,21]. Vpu also has multiple mechanisms to counteract BST-2/tetherin, and so differing degrees of partial antagonism often remain when one of the mechanisms is blocked [23,55]. Even in the βTrCP knockout cells, there does remain a very slight degree of BST-2/tetherin antagonism, but it falls below statistical significance when BST-2/tetherin levels increase beyond 10 ng. Thus, the βTrCP-independent mechanism would seem to be most relevant when BST-2/tetherin is expressed at low levels. In support of this, it was demonstrated previously that treatment of cells with MLN4924 to block Cullin ubiquitin ligases had greater impact on Vpu antagonism of BST-2/tetherin if cells were first treated with interferon, thus upregulating the BST-2/tetherin levels [27]. In addition to BST-2/tetherin levels, the level of βTrCP expression may also affect the potency of Vpu activity against BST-2/tetherin. When we reintroduced βTrCP-2 into cells where both genes had been knocked out the activity of Vpu against both CD4 and BST-2/tetherin was enhanced. Thus, the mechanism Vpu utilizes to counteract BST-2/tetherin likely varies depending on the levels of BST-2/tetherin and of βTrCP.

Finally, it should be noted that Vpu maintains at least a slight amount of activity against all three targets, (CD4, GaLV Env, and BST-2/tetherin) in the βTrCP-1/2 KO cell line. It cannot be ruled out that this cell line maintained some βTrCP-2 activity. The knockout cell line we generated targeted the third of seven of its WD40 domains that are required for interacting with its targets. Notably, we did not succeed in producing a homozygous knockout with a gRNA that targeted further upstream, and it has been reported that βTrCP-2 (FBXW11) is essential for optimal proliferation [56].
